# Crystal structure of 2,5-dimethyl-3-(2-methyl­phenyl­sulfin­yl)-1-benzo­furan

**DOI:** 10.1107/S2056989015012773

**Published:** 2015-07-08

**Authors:** Hong Dae Choi, Uk Lee

**Affiliations:** a Department of Chemistry, Dongeui University, San 24 Kaya-dong, Busanjin-gu, Busan 614-714, Republic of Korea; b Department of Chemistry, Pukyong National University, 599-1 Daeyeon 3-dong, Nam-gu, Busan 608-737, Republic of Korea

**Keywords:** crystal structure, benzo­furan, 2-methyl­phen­yl, C—H⋯O hydrogen bonds

## Abstract

In the title compound, C_17_H_16_O_2_S, the dihedral angle between the benzo­furan ring system [r.m.s. deviation = 0.009 (1) Å] and the 2-methyl­phenyl ring is 86.72 (4)°. In the crystal, weak C—H⋯O hydrogen bonds link the mol­ecules into columns along the *b*-axis direction.

## Related literature   

For the pharmacological properties of benzo­furan compounds, see: Aslam *et al.* (2009 ▸); Galal *et al.* (2009 ▸); Howlett *et al.* (1999 ▸); Wahab Khan *et al.* (2005 ▸); Ono *et al.* (2002 ▸). For natural products with a benzo­furan ring, see: Akgul & Anil (2003 ▸); Soekamto *et al.* (2003 ▸). For a related structure, see: Choi *et al.* (2012 ▸). For further synthetic details, see: Choi *et al.* (1999 ▸).
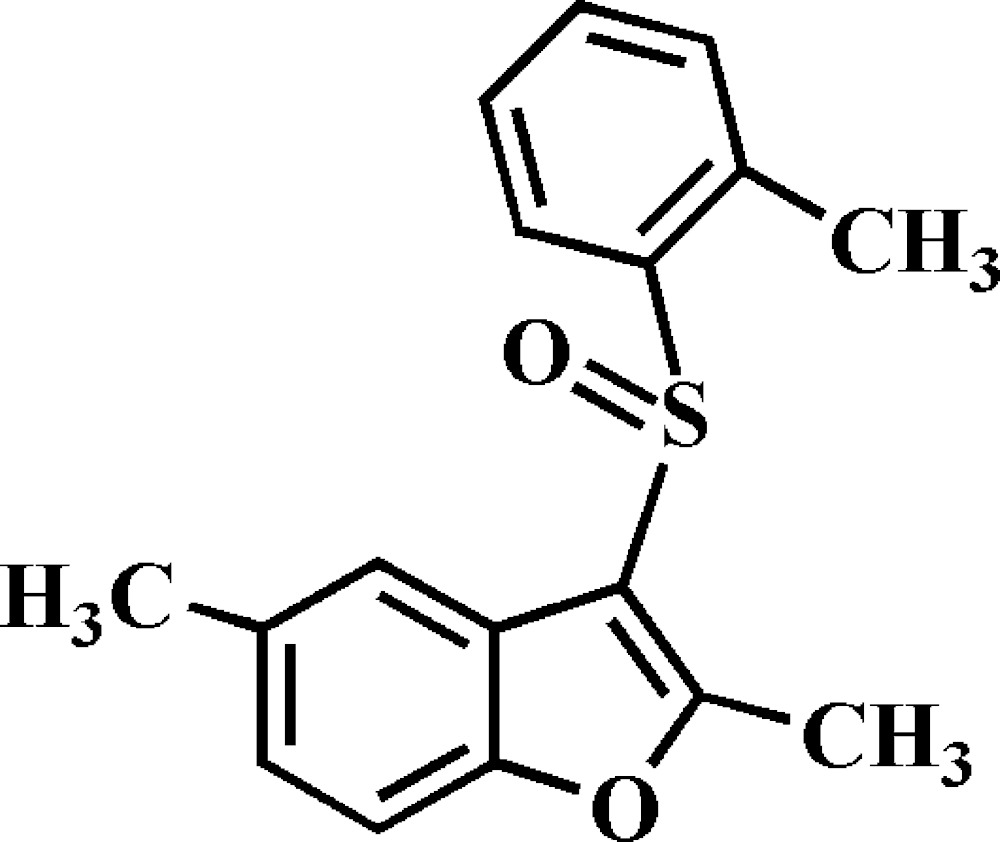


## Experimental   

### Crystal data  

C_17_H_16_O_2_S
*M* *_r_* = 284.36
Monoclinic, 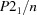

*a* = 10.8458 (2) Å
*b* = 8.0139 (1) Å
*c* = 16.4295 (2) Å
β = 96.709 (1)°
*V* = 1418.23 (4) Å^3^
*Z* = 4
Mo *K*α radiation
μ = 0.23 mm^−1^
*T* = 173 K
0.44 × 0.33 × 0.30 mm

### Data collection  

Bruker SMART APEXII CCD diffractometer
Absorption correction: multi-scan (*SADABS*; Bruker, 2009 ▸) *T*_min_ = 0.692, *T*_max_ = 0.746
25176 measured reflections
3529 independent reflections
3091 reflections with *I* > 2σ(*I*)
*R*_int_ = 0.033

### Refinement  

*R*[*F* ^2^ > 2σ(*F* ^2^)] = 0.037
*wR*(*F* ^2^) = 0.103
*S* = 1.05
3529 reflections
184 parameters
H-atom parameters constrained
Δρ_max_ = 0.30 e Å^−3^
Δρ_min_ = −0.27 e Å^−3^

### 

Data collection: *APEX2* (Bruker, 2009 ▸); cell refinement: *SAINT* (Bruker, 2009 ▸); data reduction: *SAINT*; program(s) used to solve structure: *SHELXS2014* (Sheldrick, 2008 ▸); program(s) used to refine structure: *SHELXL2014* (Sheldrick, 2015 ▸); molecular graphics: *ORTEP-3* for Windows (Farrugia, 2012 ▸) and *DIAMOND* (Brandenburg, 1998 ▸); software used to prepare material for publication: *SHELXL2014* (Sheldrick, 2008 ▸).

## Supplementary Material

Crystal structure: contains datablock(s) I. DOI: 10.1107/S2056989015012773/cv5492sup1.cif

Structure factors: contains datablock(s) I. DOI: 10.1107/S2056989015012773/cv5492Isup2.hkl

Supporting information file. DOI: 10.1107/S2056989015012773/cv5492Isup3.cml

**Fig. 1.**
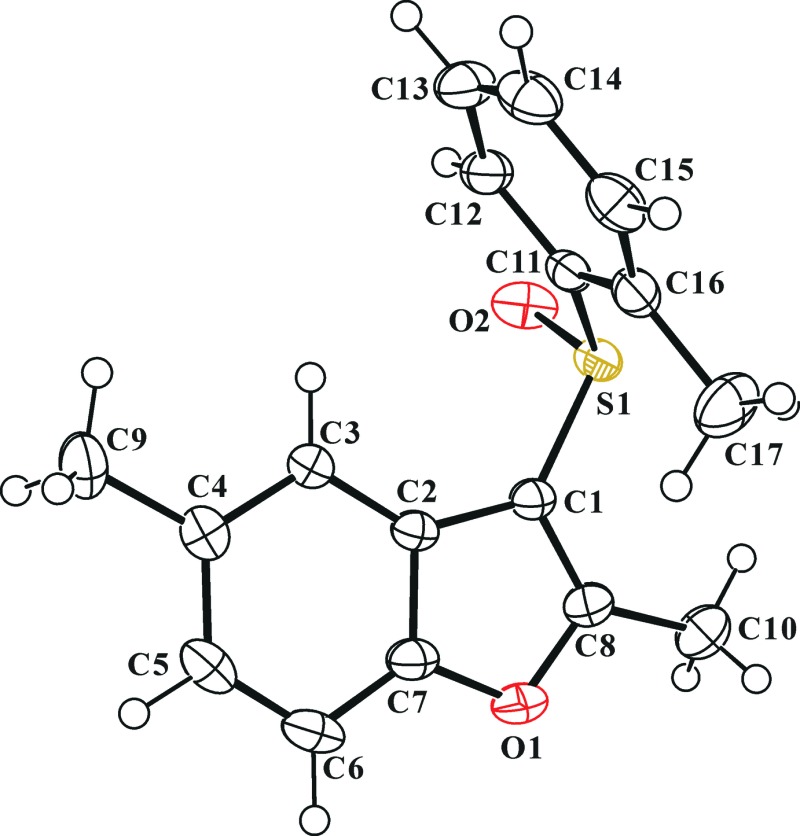
The molecular structure of the title compound, showing the atom-numbering scheme. Displacement ellipsoids are drawn at the 50% probability level. H atoms are presented as small spheres of arbitrary radius.

. DOI: 10.1107/S2056989015012773/cv5492fig1.tif
The mol­ecular structure of the title compound, showing the atom-numbering scheme. Displacement ellipsoids are drawn at the 50% probability level. H atoms are presented as small spheres of arbitrary radius.

**Fig. 2.**
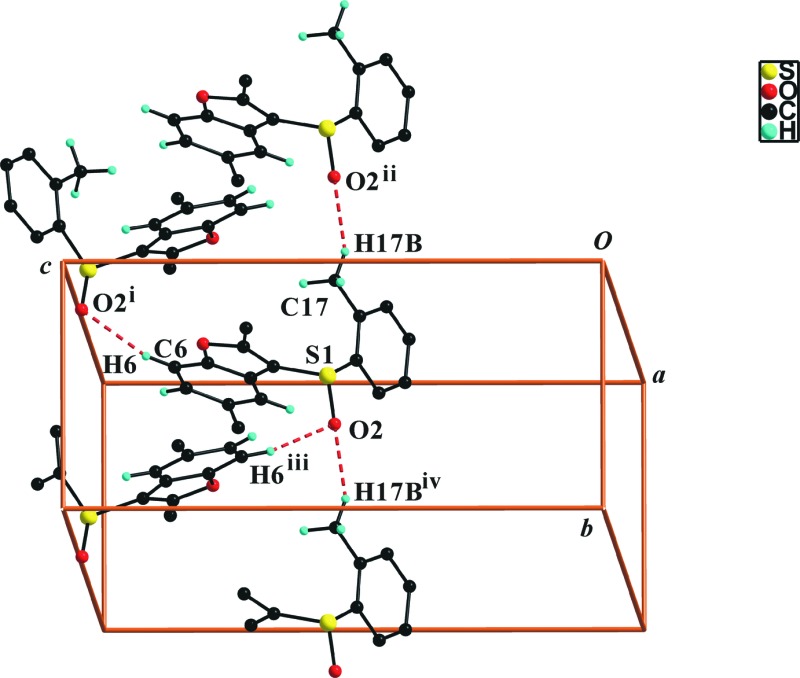
A view of the C—H···O hydrogen bonds (dotted lines) in the crystal structure of the title compound. H atoms non-participating in hydrogen-bonding were omitted for clarity. [Symmetry codes: (i) - x + 1/2, y - 1/2 , - z + 3/2; (ii) x, y - 1, z; (iii) - x + 1/2, y + 1/2 , - z + 3/2; (iv) x, y + 1, z.]

Click here for additional data file.x y z x y z x y z x y z . DOI: 10.1107/S2056989015012773/cv5492fig2.tif
A view of the C—H⋯O hydrogen bonds (dotted lines) in the crystal structure of the title compound. H atoms non-participating in hydrogen-bonding were omitted for clarity. [Symmetry codes: (i) − *x* + 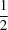, *y* − 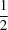, − *z* + 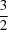; (ii) *x*, *y* − 1, *z*; (iii) − *x* + 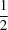, *y* + 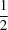, − *z* + 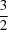; (iv) *x*, *y* + 1, *z*.]

CCDC reference: 1410058

Additional supporting information:  crystallographic information; 3D view; checkCIF report

## Figures and Tables

**Table 1:** Hydrogen-bond geometry (, )

*D*H*A*	*D*H	H*A*	*D* *A*	*D*H*A*
C6H6O2^i^	0.95	2.45	3.3772(18)	166
C17H17*B*O2^ii^	0.98	2.55	3.458(2)	154

Symmetry codes: (i) 
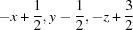
; (ii) 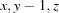.

## supplementary crystallographic information

### S1. Comment

Benzofuran derivatives show interesting pharmacological properties such as antibacterial and antifungal, antitumor and antiviral, antimicrobial activities (Aslam *et al.* 2009; Galal *et al.*, 2009; Wahab Khan *et al.*, 2005), and potential inhibitor of β-amyloid aggregation (Howlett *et al.*, 1999; Ono *et al.*, 2002). These benzofuran compounds occur in a great number of natural products. (Akgul & Anil, 2003; Soekamto *et al.*, 2003). As a part of our continuing project on benzofuran derivatives (Choi *et al.*, 2012), we report herein on the crystal structure of the title compound.

In the title molecule (Fig. 1), the benzofuran unit is essentially planar, with a mean deviation of 0.009 (1) Å from the least-squares plane defined by the nine constituent atoms. The 2-methylphenyl ring is essentially planar, with a mean deviation of 0.004 (1) Å from the least-squares plane defined by the six constituent atoms. The dihedral angle formed by the benzofuran ring and the 2-methylphenyl ring is 86.72 (4)°. In the crystal, molecules are linked into a chain along the *b* axis direction by C—H···O hydrogen bonds (Table 1 and Fig. 2). These molecules are connected on either side of this chain by further C—H···O hydrogen bonds (Table 1 and Fig. 2).

### S2. Experimental

The starting material 2,5-dimethyl-3-(2-methylphenylsulfanyl)-1-benzofuran was prepared by literature method (Choi *et al.* 1999). 3-Chloroperoxybenzoic acid (77%, 224 mg, 1.0 mmol) was added in small portions to a stirred solution of 2,5-dimethyl-3-(2-methylphenylsulfanyl)-1-benzofuran (241 mg, 0.9 mmol) in dichloromethane (25 ml) at 273 K. After being stirred at room temperature for 8h, the mixture was washed with saturated sodium bicarbonate solution (2 X 10 ml) and the organic layer was separated, dried over magnesium sulfate, filtered and concentrated at reduced pressure. The residue was purified by column chromatography (hexane–ethyl acetate, 2:1 *v*/*v*) to afford the title compound as a colorless solid [yield 68% (174 mg); m.p. 415–416 K; *R*_f_ = 0.49 (hexane–ethyl acetate, 2:1 *v*/*v*)]. Single crystals suitable for X-ray diffraction were prepared by slow evaporation of a solution of the title compound (21 mg) in ethyl acetate (20 ml) at room temperature.

### S3. Refinement

All H atoms were positioned geometrically and refined using a riding model, with C—H = 0.95 Å for aryl and 0.98 Å for methyl H atoms, *U*_iso_ (H) = 1.2*U*_eq_ (C) for aryl and 1.5*U*_eq_ (C)) for methyl H atoms. The positions of methyl hydrogens were optimized using the command AFIX in *SHELXL-2014/7* (Sheldrick, 2015)

### Crystal data

**Table:**

C_17_H_16_O_2_S	*F*(000) = 600
*M**_r_* = 284.36	*D*_x_ = 1.332 Mg m^−^^3^
Monoclinic, *P*2_1_/*n*	Mo *K*α radiation, λ = 0.71073 Å
*a* = 10.8458 (2) Å	Cell parameters from 9125 reflections
*b* = 8.0139 (1) Å	θ = 2.5–28.1°
*c* = 16.4295 (2) Å	µ = 0.23 mm^−^^1^
β = 96.709 (1)°	*T* = 173 K
*V* = 1418.23 (4) Å^3^	Block, colourless
*Z* = 4	0.44 × 0.33 × 0.30 mm

### Data collection

**Table:**

Bruker SMART APEXII CCD diffractometer	3529 independent reflections
Radiation source: rotating anode	3091 reflections with *I* > 2σ(*I*)
Graphite multilayer monochromator	*R*_int_ = 0.033
Detector resolution: 10.0 pixels mm^-1^	θ_max_ = 28.4°, θ_min_ = 2.1°
φ and ω scans	*h* = −14→14
Absorption correction: multi-scan (*SADABS*; Bruker, 2009)	*k* = −10→10
*T*_min_ = 0.692, *T*_max_ = 0.746	*l* = −21→20
25176 measured reflections	

### Refinement

**Table:**

Refinement on *F*^2^	Primary atom site location: structure-invariant direct methods
Least-squares matrix: full	Secondary atom site location: difference Fourier map
*R*[*F*^2^ > 2σ(*F*^2^)] = 0.037	Hydrogen site location: difference Fourier map
*wR*(*F*^2^) = 0.103	H-atom parameters constrained
*S* = 1.05	*w* = 1/[σ^2^(*F*_o_^2^) + (0.0515*P*)^2^ + 0.5244*P*] where *P* = (*F*_o_^2^ + 2*F*_c_^2^)/3
3529 reflections	(Δ/σ)_max_ = 0.001
184 parameters	Δρ_max_ = 0.30 e Å^−^^3^
0 restraints	Δρ_min_ = −0.27 e Å^−^^3^

### Special details

**Table:**

Experimental. ^1^H NMR (δ p.p.m., CDCl_3_, 400 Hz): 8.35 (*d, J* = 7.88 Hz, 1H), 7.53–7.57 (m, 1H), 7.38–7.42 (m, 1H), 7.26 (*d, J* = 7.02 Hz, 1H), 7.15 (*d, J* = 7.52 Hz, 1H), 6.98–7.03 (m, 2H), 2.73 (s, 3H), 2.25 (s, 3H), 2.13 (s, 3H),
Geometry. All esds (except the esd in the dihedral angle between two l.s. planes) are estimated using the full covariance matrix. The cell esds are taken into account individually in the estimation of esds in distances, angles and torsion angles; correlations between esds in cell parameters are only used when they are defined by crystal symmetry. An approximate (isotropic) treatment of cell esds is used for estimating esds involving l.s. planes.

### Fractional atomic coordinates and isotropic or equivalent isotropic displacement parameters (Å^2^)

**Table:**

	*x*	*y*	*z*	*U*_iso_*/*U*_eq_	
S1	0.17973 (3)	0.37549 (4)	0.52324 (2)	0.02675 (11)	
O1	0.17654 (10)	0.24730 (14)	0.75315 (6)	0.0349 (2)	
O2	0.21361 (11)	0.55407 (13)	0.51308 (6)	0.0381 (3)	
C1	0.21664 (12)	0.31930 (17)	0.62666 (8)	0.0250 (3)	
C2	0.33439 (12)	0.30795 (16)	0.67783 (8)	0.0236 (3)	
C3	0.45906 (12)	0.33131 (16)	0.66789 (8)	0.0255 (3)	
H3	0.4825	0.3619	0.6160	0.031*	
C4	0.54881 (13)	0.30933 (18)	0.73475 (9)	0.0307 (3)	
C5	0.51194 (15)	0.2614 (2)	0.81059 (9)	0.0363 (3)	
H5	0.5740	0.2456	0.8557	0.044*	
C6	0.38937 (16)	0.23627 (19)	0.82225 (9)	0.0355 (3)	
H6	0.3656	0.2031	0.8737	0.043*	
C7	0.30345 (13)	0.26225 (18)	0.75471 (8)	0.0287 (3)	
C8	0.12638 (13)	0.28335 (19)	0.67489 (9)	0.0304 (3)	
C9	0.68418 (15)	0.3366 (2)	0.72713 (11)	0.0434 (4)	
H9A	0.6941	0.3692	0.6708	0.065*	
H9B	0.7301	0.2331	0.7408	0.065*	
H9C	0.7165	0.4252	0.7648	0.065*	
C10	−0.01086 (15)	0.2761 (3)	0.65979 (11)	0.0452 (4)	
H10A	−0.0380	0.3087	0.6030	0.068*	
H10B	−0.0466	0.3527	0.6972	0.068*	
H10C	−0.0388	0.1622	0.6691	0.068*	
C11	0.29473 (12)	0.25559 (17)	0.47850 (7)	0.0236 (3)	
C12	0.38520 (14)	0.34425 (18)	0.44408 (8)	0.0297 (3)	
H12	0.3893	0.4622	0.4493	0.036*	
C13	0.46993 (15)	0.2599 (2)	0.40181 (9)	0.0365 (3)	
H13	0.5331	0.3197	0.3788	0.044*	
C14	0.46160 (15)	0.0895 (2)	0.39362 (9)	0.0363 (3)	
H14	0.5183	0.0315	0.3639	0.044*	
C15	0.37125 (14)	0.00203 (19)	0.42834 (9)	0.0338 (3)	
H15	0.3675	−0.1159	0.4225	0.041*	
C16	0.28551 (13)	0.08234 (18)	0.47168 (8)	0.0285 (3)	
C17	0.18974 (17)	−0.0156 (2)	0.50997 (12)	0.0464 (4)	
H17A	0.2083	−0.0119	0.5698	0.070*	
H17B	0.1907	−0.1318	0.4914	0.070*	
H17C	0.1075	0.0326	0.4938	0.070*	

### Atomic displacement parameters (Å^2^)

**Table:**

	*U*^11^	*U*^22^	*U*^33^	*U*^12^	*U*^13^	*U*^23^
S1	0.02608 (18)	0.03273 (19)	0.02139 (17)	0.00435 (12)	0.00261 (12)	0.00056 (12)
O1	0.0357 (6)	0.0446 (6)	0.0266 (5)	−0.0031 (5)	0.0128 (4)	0.0039 (4)
O2	0.0555 (7)	0.0288 (5)	0.0317 (5)	0.0098 (5)	0.0130 (5)	0.0029 (4)
C1	0.0253 (6)	0.0285 (6)	0.0219 (6)	0.0001 (5)	0.0053 (5)	0.0000 (5)
C2	0.0290 (6)	0.0229 (6)	0.0194 (6)	0.0002 (5)	0.0046 (5)	−0.0003 (4)
C3	0.0266 (6)	0.0280 (6)	0.0220 (6)	−0.0007 (5)	0.0026 (5)	−0.0005 (5)
C4	0.0314 (7)	0.0304 (7)	0.0292 (7)	0.0008 (5)	−0.0014 (6)	−0.0019 (5)
C5	0.0433 (8)	0.0393 (8)	0.0238 (7)	0.0031 (6)	−0.0064 (6)	0.0009 (6)
C6	0.0515 (9)	0.0353 (8)	0.0198 (6)	0.0018 (7)	0.0052 (6)	0.0032 (5)
C7	0.0331 (7)	0.0312 (7)	0.0230 (6)	−0.0009 (5)	0.0084 (5)	0.0008 (5)
C8	0.0289 (7)	0.0362 (7)	0.0275 (7)	−0.0020 (5)	0.0089 (5)	−0.0005 (5)
C9	0.0293 (8)	0.0558 (10)	0.0424 (9)	−0.0019 (7)	−0.0066 (7)	−0.0019 (7)
C10	0.0281 (8)	0.0646 (11)	0.0451 (9)	−0.0056 (7)	0.0132 (7)	−0.0002 (8)
C11	0.0245 (6)	0.0286 (7)	0.0171 (5)	0.0019 (5)	0.0003 (5)	−0.0007 (4)
C12	0.0344 (7)	0.0309 (7)	0.0249 (6)	−0.0023 (5)	0.0073 (5)	−0.0004 (5)
C13	0.0363 (8)	0.0450 (9)	0.0304 (7)	0.0001 (6)	0.0128 (6)	0.0006 (6)
C14	0.0373 (8)	0.0457 (9)	0.0263 (7)	0.0124 (7)	0.0049 (6)	−0.0039 (6)
C15	0.0418 (8)	0.0299 (7)	0.0285 (7)	0.0069 (6)	−0.0015 (6)	−0.0039 (5)
C16	0.0313 (7)	0.0300 (7)	0.0232 (6)	−0.0014 (5)	−0.0011 (5)	−0.0005 (5)
C17	0.0526 (10)	0.0321 (8)	0.0570 (11)	−0.0126 (7)	0.0168 (8)	−0.0016 (7)

### Geometric parameters (Å, º)

**Table:**

S1—O2	1.4918 (11)	C9—H9B	0.9800
S1—C1	1.7580 (13)	C9—H9C	0.9800
S1—C11	1.7982 (13)	C10—H10A	0.9800
O1—C8	1.3671 (18)	C10—H10B	0.9800
O1—C7	1.3789 (17)	C10—H10C	0.9800
C1—C8	1.3604 (18)	C11—C12	1.3846 (19)
C1—C2	1.4476 (18)	C11—C16	1.3955 (19)
C2—C7	1.3932 (17)	C12—C13	1.390 (2)
C2—C3	1.3934 (18)	C12—H12	0.9500
C3—C4	1.3912 (19)	C13—C14	1.375 (2)
C3—H3	0.9500	C13—H13	0.9500
C4—C5	1.406 (2)	C14—C15	1.381 (2)
C4—C9	1.504 (2)	C14—H14	0.9500
C5—C6	1.380 (2)	C15—C16	1.393 (2)
C5—H5	0.9500	C15—H15	0.9500
C6—C7	1.379 (2)	C16—C17	1.498 (2)
C6—H6	0.9500	C17—H17A	0.9800
C8—C10	1.481 (2)	C17—H17B	0.9800
C9—H9A	0.9800	C17—H17C	0.9800
			
O2—S1—C1	108.82 (6)	H9A—C9—H9C	109.5
O2—S1—C11	105.97 (6)	H9B—C9—H9C	109.5
C1—S1—C11	99.66 (6)	C8—C10—H10A	109.5
C8—O1—C7	106.61 (10)	C8—C10—H10B	109.5
C8—C1—C2	107.12 (12)	H10A—C10—H10B	109.5
C8—C1—S1	121.27 (11)	C8—C10—H10C	109.5
C2—C1—S1	131.55 (10)	H10A—C10—H10C	109.5
C7—C2—C3	118.78 (12)	H10B—C10—H10C	109.5
C7—C2—C1	104.68 (11)	C12—C11—C16	121.69 (12)
C3—C2—C1	136.54 (12)	C12—C11—S1	116.82 (10)
C4—C3—C2	119.30 (13)	C16—C11—S1	121.19 (10)
C4—C3—H3	120.3	C11—C12—C13	119.74 (14)
C2—C3—H3	120.3	C11—C12—H12	120.1
C3—C4—C5	119.29 (13)	C13—C12—H12	120.1
C3—C4—C9	121.04 (14)	C14—C13—C12	119.48 (14)
C5—C4—C9	119.67 (14)	C14—C13—H13	120.3
C6—C5—C4	122.77 (13)	C12—C13—H13	120.3
C6—C5—H5	118.6	C13—C14—C15	120.36 (14)
C4—C5—H5	118.6	C13—C14—H14	119.8
C7—C6—C5	115.96 (13)	C15—C14—H14	119.8
C7—C6—H6	122.0	C14—C15—C16	121.67 (14)
C5—C6—H6	122.0	C14—C15—H15	119.2
O1—C7—C6	125.53 (13)	C16—C15—H15	119.2
O1—C7—C2	110.59 (12)	C15—C16—C11	117.05 (13)
C6—C7—C2	123.88 (14)	C15—C16—C17	120.63 (14)
C1—C8—O1	110.99 (12)	C11—C16—C17	122.31 (13)
C1—C8—C10	133.48 (14)	C16—C17—H17A	109.5
O1—C8—C10	115.53 (12)	C16—C17—H17B	109.5
C4—C9—H9A	109.5	H17A—C17—H17B	109.5
C4—C9—H9B	109.5	C16—C17—H17C	109.5
H9A—C9—H9B	109.5	H17A—C17—H17C	109.5
C4—C9—H9C	109.5	H17B—C17—H17C	109.5

### Hydrogen-bond geometry (Å, º)

**Table:**

*D*—H···*A*	*D*—H	H···*A*	*D*···*A*	*D*—H···*A*
C6—H6···O2^i^	0.95	2.45	3.3772 (18)	166
C17—H17*B*···O2^ii^	0.98	2.55	3.458 (2)	154

Symmetry codes: (i) −*x*+1/2, *y*−1/2, −*z*+3/2; (ii) *x*, *y*−1, *z*.
