# Supplementary figures and images for: Crystal structure of 2,5-dimethyl-3-(2-methyl­phenyl­sulfin­yl)-1-benzo­furan
Source: Acta Crystallogr E Crystallogr Commun. 2015 Jul 8;71(Pt 8):o554–5. doi: 10.1107/S2056989015012773 (PMC4571393; doi:10.1107/S2056989015012773)

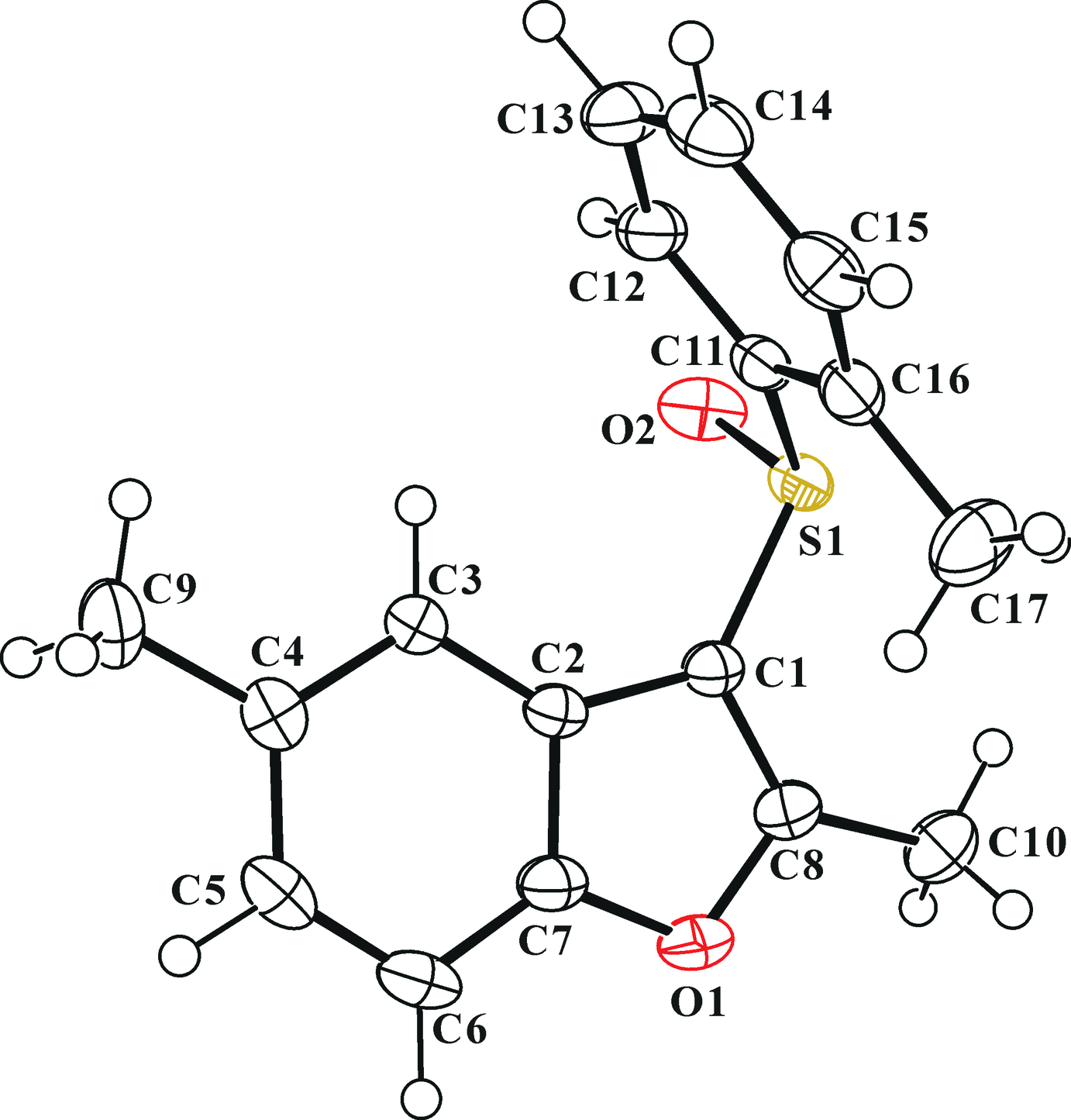

Supplement: Supplementary file 4 [file e-71-0o554-fig1.tif]

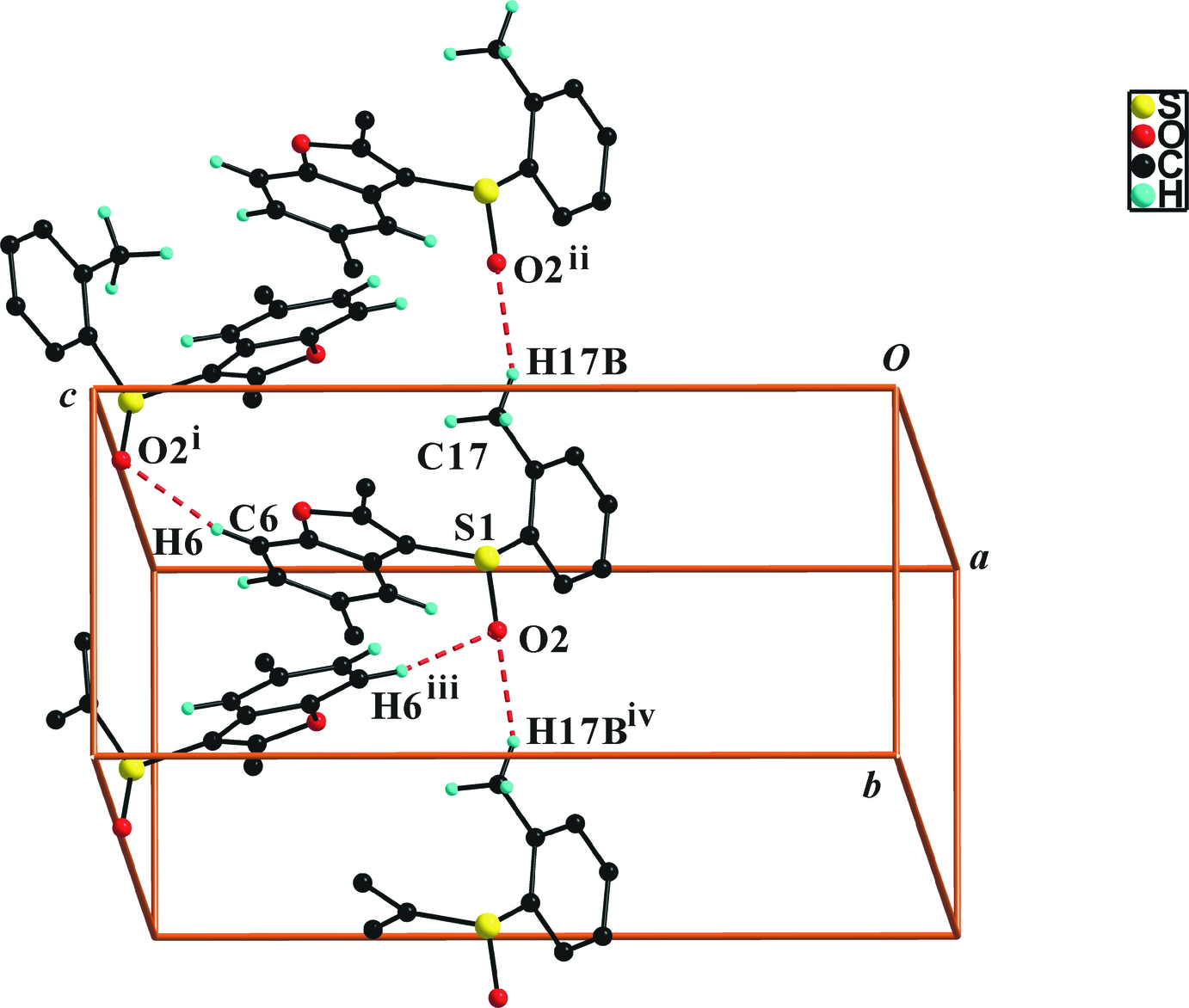

Supplement: Supplementary file 5 [file e-71-0o554-fig2.tif]
